# High throughput sample processing and automated scoring

**DOI:** 10.3389/fgene.2014.00373

**Published:** 2014-10-28

**Authors:** Gunnar Brunborg, Petra Jackson, Sergey Shaposhnikov, Hildegunn Dahl, Amaya Azqueta, Andrew R. Collins, Kristine B. Gutzkow

**Affiliations:** ^1^Department of Chemicals and Radiation, Division of Environmental Medicine, Norwegian Institute of Public HealthOslo, Norway; ^2^National Research Centre for the Working EnvironmentCopenhagen, Denmark; ^3^Department of Nutrition, University of OsloOslo, Norway; ^4^NorGenoTech AS, SkreiaNorway; ^5^Department of Pharmacology and Toxicology, University of NavarraPamplona, Spain

**Keywords:** high throughput, comet assay, genotoxicity, *in vivo* sample processing, automated scoring

## Abstract

The comet assay is a sensitive and versatile method for assessing DNA damage in cells. In the traditional version of the assay, there are many manual steps involved and few samples can be treated in one experiment. High throughput (HT) modifications have been developed during recent years, and they are reviewed and discussed. These modifications include accelerated scoring of comets; other important elements that have been studied and adapted to HT are cultivation and manipulation of cells or tissues before and after exposure, and freezing of treated samples until comet analysis and scoring. HT methods save time and money but they are useful also for other reasons: large-scale experiments may be performed which are otherwise not practicable (e.g., analysis of many organs from exposed animals, and human biomonitoring studies), and automation gives more uniform sample treatment and less dependence on operator performance. The HT modifications now available vary largely in their versatility, capacity, complexity, and costs. The bottleneck for further increase of throughput appears to be the scoring.

## INTRODUCTION

The comet assay in its basic form is a sensitive and relatively simple assay requiring little instrumentation. The original method (Ostling and Johanson, 1984) was later improved and standardized by Tice and co-workers (Singh et al., 1988). It involved up to three layers of agarose on a glass slide: a support gel, to which the mixture of agarose and cells is added as a second layer, and then a cover gel. Each layer needs a glass coverslip which is then removed once the gel has set. These operations cannot easily be automated. In recent years, various simplifications and also modified protocols have been presented, e.g., reducing the number of gel layers from three to one, introducing other substrates and formats than glass microscope slides, and skipping the coverslip entirely. These revisions make the assay more amenable to automation and high throughput (HT). Other innovations relate not only to the analysis part of the comet assay, but also to those elements consisting of cell cultivation, *in vitro* exposures to genotoxicants, as well as processing and storage of samples from *in vivo* exposed animals. Various forms of HT methods have appeared, with the potential to increase the number of samples that can be treated in one experiment from a maximum of ∼40 in 1 day, to at least 1,200 (Gutzkow et al., 2013). This is useful since it saves time and money, but also since other types of experiments are possible, such as the analysis of multiple tissues from animals exposed *in vivo* and large biomonitoring studies.

A protocol for the *in vivo* comet is now close to being approved by OECD (OECD 2013; Pant et al., 2014). Furthermore, European Food Safety Authority (EFSA) has issued guidance on the minimum requirements for the *in vivo* comet assay (EFSA, 2012). However, these protocols do not include or discuss HT modifications. The EC Regulation on chemicals and their safe use, REACH, obliges the chemical industry to test chemicals produced at volumes above 100 tons per year for toxic effects on health and the environment – a task that would benefit from HT methods. Hardly any alternatives to the comet assay are available for the detection of genotoxicity in specific organs *in vivo*: mutation analysis with transgenic rodents (TGR) is costly and requires specific strains of animals (OECD, 2013). There is therefore an obvious need for a reliable and validated HT comet assay preferably with some degree of automation.

We here review the most relevant HT comet assay systems, all of which have appeared during the last 15 years, and we briefly discuss their main features. We also discuss some methodological approaches which need further evaluation and do not yet offer an increased throughput but which nevertheless seem to have a potential for HT.

## COMET ASSAY MODIFICATIONS

Much emphasis has been placed on avoiding the laborious two or three layer agarose gel sandwich and the use of coverslips. Several routes have been followed: (i) Modifications of glass formats; (ii) polyester films to replace glass slides; (iii) microtitre wells used for cell growth and gel forming; and (iv) more advanced technologies including cell microarrays, and microfluidics.

### MINIGELS ON GLASS SLIDES

Collins and coworkers (Shaposhnikov et al., 2010) developed a format based on minigels separated on a standard glass slide by means of a silicone gasket clamped to the slide, using a tailor-made aluminum/plastic holder (see Figure 1 in Shaposhnikov et al., 2010). Cell-agarose samples are added to each of the 12 wells and may be subjected to different lesion-specific endonucleases or other specific treatments such as fluorescent *in situ* hybridization with different DNA-probes for staining, using the same slide. In addition, the unit has a special application in studies of DNA repair capacity of cell extracts. Most recently the 12-gel glass slide format was used in a method designed for assessing BER and NER repair capacities in frozen tissues from cancer patients and healthy controls (Slyskova et al., 2014). Most electrophoresis tanks hold 10–20 slides, and so running a few 100 samples in one experiment is well within reach.

### MORE SAMPLES ON A GLASS SURFACE

The first Comet assay commercial kit was described by Lemay and Wood (1999). Areas on a glass slide treated with a proprietary technology provide immobilization of gel samples, and there are hydrophobic spacers to separate neighboring samples (Trevigen CometSlide^TM^; Trevigen Inc., Gaithersburg, MD, USA). Samples are added manually and the glass plates are treated and electrophoresed in the same way as in the traditional assay. More recently, larger glass slides are offered from the same supplier holding 96 samples. The slides are rather expensive and add significantly to the total cost of the assay, although the smaller size of the gels reduces the consumption of chemicals. A format of 4 ×5 = 20 samples has been used in some studies (Reelfs et al., 2011; Yuan et al., 2012; Jackson et al., 2013), but the glass formats (at least the ×96 version) do not seem to have been subjected to a systematic validation vs. the standard method. Jackson et al. (2013) recently successfully adapted the ×20 Trevigen glass slides to automated scoring by means of IMSTAR^TM^ Pathfinder.

Ritter and Knebel (2009) described a system involving 20 samples spotted onto glass slides (prototype comet slide, patent pending). Scoring was with an automated system developed by the authors; its principles and function were described in some detail. No further information was provided on the software and potential availability for other comet users. The authors describe the high reproducibility of the system and it is argued that it is suitable for higher throughput genotoxicity testing.

An increase in the number of samples per glass slide has also been described by Zhang et al. (2011) who used a plastic device to spread out five or more 20 μL samples on each standard glass slide (with no coverslips). The method was used to study effects of one chemical (melatonin) on the repair of DNA damage induced by UV-B in *Gentiana* protoplasts. The authors claim that the sensitivity is retained compared to the conventional assay and that it is easy to use. However, no further validation was reported.

### POLYESTER FILMS REPLACE GLASS

McNamee et al. (2000) were the first to describe how comet assay agarose gels could be attached to a coated polyester film, thus replacing the glass slide. The GelBond® film is a thin unbreakable film used as a support for agarose gels in general. Twelve square gels are molded per film by means of plastic frames (SuperCell chambers), with no coverslips, and four films may be electrophoresed together in one tank. The method was validated using hydrogen peroxide and ionizing radiation, and the results for sensitivity were similar to those reported for the traditional assay. After scoring, the dried films may be stored securely, requiring little space. After its first publication, the method has been used in a number of laboratories. We subsequently replaced the disposable SuperCell chambers with a brass plate with cylindrical openings lined with Teflon, allowing 12 round samples (each of 30–70 μL) to be added to one GelBond® film of size 70 ×90 mm (Hertel-Aas et al., 2011). However, the number of samples was still rather modest (48 samples per electrophoresis).

We recently took the polyester film technology further, to accommodate up to 96 minigels on one Gelbond® film in a 96-well format, but with no use of molds, wells, or separating surfaces (Gutzkow et al., 2013). This was possible, since – with a small volume of gel (3–6 uL) – a droplet added to the cold film surface forms a uniform lens-shaped disc (see Figure 2 in Gutzkow et al., 2013). The agarose/cell samples are applied with a multi-pipette; a template is used to position the center of each sample. Such samples (minigels) settle within seconds on a cold surface. [Ostling and Johanson (1984) termed their technique *a microelectrophoretic study*, whereas Tice and Singh (Singh et al., 1988) used the expression *microgels*; our gel samples are of microliter size and indeed much smaller, but we use the term minigels since they are not of the micro scale which is now often used in molecular gel electrophoresis.] The film, previously cut to the size of a standard microtiter plate format, is at all stages of the comet assay attached to a plastic frame for ease of manipulation and to protect the gels (see Figure 2 in Gutzkow et al., 2013). We have processed 1200 samples in parallel in three electrophoresis tanks each holding four films. Processing (per sample) takes in total (but excluding scoring) 5–10 times less time than with glass slides (Gutzkow et al., 2013). The system has been validated using ionizing radiation to induce defined numbers of DNA strand breaks per cell, and it was verified that the 96-minigel format has the same sensitivity and dynamic range for detecting DNA damage as the standard assay based on glass slides (see also McNamee et al., 2000). For detection of base damage, parallel films are immersed in appropriate DNA repair endonuclease solutions, such as formamidopyrimidine DNA glycosylase for oxidized purines, or denV (T4 endonuclease V) for UV-induced damage. A silicone gasket and a bottomless microtiter well plate can be used to treat individual samples with chemicals or enzymes in much the same way as with the glass minigel system (Shaposhnikov et al., 2010). Scoring is done either with a semi-automated system (Comet Assay IV, Perceptive Instruments, Bury St. Edmunds, Suffolk, UK), or with the fully automated system of Imstar Pathfinder^TM^ MLA (Paris, France). This is a simple, versatile, and low-cost HT format, which we have used with a variety of cell types and tissues. Of particular importance is that the samples never fall off the film surface, even after extended lysis times (weeks) which are sometimes needed for logistic reasons (for example when preparing cell samples from fish in the open sea, for subsequent comet assay analysis; personal communication, Professor Ketil Hylland, University of Oslo). Robotic application of samples can be used to achieve precise positions of samples facilitating automated scoring. The minigel system is amenable to full automation of all steps, including addition of samples and processing of films.

### ADVANCED METHODOLOGIES

Several more advanced formats have appeared in recent years. Stang and Witte (2009) developed a special 96-well multi-chamber with an agarose-containing bottom plate to which cells are attached; they may be cultured and also exposed in these wells. The multi-chamber integrates a viability assay which gives valuable information on cell status prior to comet assay. After cell treatment, the bottom plate is detached from the chamber structure and undergoes standard comet assay analysis. Originally developed for adherent cells, there is no need for detaching and harvesting the cells and the comet assay can be run immediately after cell exposure. Depending on cell type, the cells need up to 16 h to attach to the multi cell-chamber plate prior to exposure. The technology was later adapted to non-adherent cells including lymphocytes (Stang and Witte, 2010) and the authors combined this system with fully automated scoring of comets (Stang et al., 2010) using MetaSystems CometImager, thereby decreasing the evaluation time for comets by a factor of 10. In 1 day 400 samples could be fully processed. Validation was performed using methyl methanesulfonate (MMS) and H_2_O_2_ treatment of cells, and results were compared with those from semiautomated systems. However, the maximum level of DNA damage used in this evaluation was relatively low (Tail% DNA not above 45%). It is our experience that automated scoring systems may be less able to accurately identify and measure heavily damaged cells, resulting in reduced sensitivity and dynamic range. Nevertheless, the system of Stang and Witte (2010) represents a substantially increased throughput, integrating cell exposure, the comet assay, and also the scoring. Although several manual operations seem to be involved, the system deserves to be named HT. We have however, not been able to identify publications from independent laboratories using these methods after their first publication.

Engelward and co-workers described a very interesting method in which single cells are trapped in an array of agarose on a GelBond® film (Wood et al., 2010). Microwells of size 19–54 μm are produced using a microfabricated stamp; cells are added and are captured in these microwells by gravity (taking 0.5–1 h), at defined numbers (1–10 cells, depending on well size). The untrapped cells are aspirated and washed off before agarose is added to fill the microwells. These arrays may be fixed to a bottomless microtiter plate, allowing specific chemical treatment of cells in each of 96 wells, either before or after adding agarose. After lysis, different enzymes or repair inhibitors can be applied to nucleoids in microwells to measure different types of DNA lesions and their repair. The technology reduces the problem of overlapping comets. Furthermore, cells are trapped in one focal plane which facilitates cell location and automated scoring. The concept, which is named CometChip, has several advantages; for example, cell aggregates were efficiently analyzed for DNA damage (Wood et al., 2010). The CometChip works both with non-adherent and adherent cells. The inventors argue that the array can be mass-produced and that the assay is simple, however, the CometChip appears to be somewhat more technically demanding than the more traditional methods, and the application of cells is also more time-consuming. Some validation of the CometChip has been reported (Weingeist et al., 2013) and it was recently used by the same authors to analyze the genotoxicity of five types of nanoparticles (Watson et al., 2014).

An *in situ* comet assay substrate was developed by Mercey et al. (2010). A three-dimensional agarose layer was covalently bound to a glass slide and micropatterned into structures of defined sizes. Polarized cells keep their polarity and their differentiated state in these structures. A micropattern of 900 μm × 900 μm was used to analyze the genotoxicity of MMS, followed by a standard comet assay analysis. Scoring took place with confocal microscopy. The results obtained with this method indicate that it is suitable for HT cytotoxicity and genotoxicity screening, with automated scoring. This is an interesting method with potential for HT testing, but it is technically demanding and probably has a long way to go to become a generally usable comet assay. The operations appear to be technologically more challenging than the other formats for HT comet analysis, and there seems to be no follow-up since the method was first presented in 2010.

Li et al. (2013) recently described a novel concept based on a microfluidic chip. A 100 channels in agarose, each of height and width 20 μm×20 μm, length 20 mm, are positioned on a single glass slide. Cells (10,000) are introduced in these channels and subjected to comet analyses more or less as in the conventional assay. Electrophoresis takes place perpendicular to the channels. Since the cells are positioned precisely along the channels, their comet tails can be analyzed efficiently. This fascinating approach has great potentials but has so far been neither validated nor developed into a standardized comet assay.

### CELL TREATMENTS AND SAMPLE PROCESSING

Efficient treatment of cell samples is an essential part of a HT assay, whether the cells are cultivated and treated *in vitro* or are derived from *in vivo* experiments. Considerable efforts have been made to design satisfactory logistics for the comet assay: The assay itself should be able to analyze large numbers of samples, but this has no value if high-quality samples cannot be processed in sufficient numbers for the subsequent HT comet analysis. For *in vitro* exposures, this problem may be overcome in different ways. For instance, Kiskinis et al. (2002) described an integrated exposure assay in which cells in one 96-well plate are treated with several test chemicals per experiment. Also cytotoxicity tests are performed in the wells, but cell samples are thereafter taken out and analyzed with a standard comet assay. This is clearly not a HT system, but the approach increased the genotoxicity testing throughput by more than twofold.

The HT comet assay is a must for analysis of multiple samples collected in large biomonitoring studies, prospective cohort studies, clinical trials, or in large-scale toxicology screening tests. All these study types involve many samples, often collected over long periods of time, sometimes at several locations. For logistical reasons, it is not always possible to analyze the samples when they are fresh, and freezing the samples is therefore an alternative. Although freezing of samples has been criticized (Azqueta and Collins, 2013), there is accumulating evidence that many tissue samples may indeed be snap-frozen and stored without compromising DNA integrity (Pant et al., 2014). Rigorous control of methods is needed to avoid introduction of spurious DNA damage during post-exposure processing. An optimized protocol for freezing and thawing cells and tissues was recently described (Jackson et al., 2013), suitable for large animal experiments. Both the freezing and the thawing may be critical for preservation of DNA integrity. Cells/tissues can be frozen directly as small sub-samples or as cell suspensions in freezing medium with 10% DMSO (Recio et al., 2012; Jackson et al., 2013), but snap-freezing of blood cells is also possible (Al-Salmani et al., 2011; Akor-Dewu et al., 2014). Frozen cell samples have been distributed as part of inter-laboratory trials (Forchhammer et al., 2010; Ersson and Moller, 2011), and frozen human whole blood or mononuclear cells are used as markers of environmental or dietary exposure (Collins et al., 1997a, 2014). In such studies, samples should be from a single bulk collection, to avoid seasonal, and lifestyle variations, again necessitating freezing (Moller et al., 2000; Slyskova et al., 2014).

Freezing multiple tissues from animal experiments should be well received by society since this often represents less use of animals (Pant et al., 2014). The cosmetic industry is not allowed to use animal testing and is in the process of developing human reconstructed skin models, and such 3-D systems are now under validation for both the micronucleus and the comet assays. The reconstructed skin comet project (part of the 3 D-skin project set up by the Cosmetics Europe Genotoxicity Task Force), based on EpiDerm^TM^ tissues, has completed the first validations (phase 1 and 2), and claims good intra- and inter-laboratory reproducibility. Several chemicals were tested with apparently good reproducibility, but the last step (phase 3) in this validation met with some challenges such as inter- and intra-laboratory variability and high background of solvent controls due to an insufficient quality of the tissue (Pfuhler et al., 2014). A need for optimizing and standardizing the protocol for tissue preparations is clearly indicated.

### SCORING OF COMETS

The need for efficient scoring methods increases dramatically with the HT systems described above. Semi-automated scoring is highly time-consuming and easily becomes the bottleneck: an average 96-spot comet assay scoring, with 30–50 comets per samples, takes at least a day to perform. Software-based methods for unattended comet scoring are now available. They increase the efficiency at least 10-fold and they avoid tiring microscope operations.

The automated systems which are available in principle perform scoring in the same way as the semi-automated scoring systems (Perceptive Instruments Comet IV; Kinet Imaging, Andor; and others) but they to the job more quickly and with little or no operator interaction. A comet is identified and focused, the image is stored, and the system performs the image analysis to determine comet tail parameters. Two commercial systems are known to us: MetaSystems CometImager, and Imstar Pathfinder^TM^. The performance of these systems has been described in some detail (Stang et al., 2010; Azqueta et al., 2011; Sharma et al., 2012). However, the speed, sensitivity, dynamic range, and need for operator intervention are still important issues. In particular, faint comets represent a challenge, since their head and tail lengths are difficult to measure. The commercial systems were originally developed for scoring comets in one or two samples on glass slides, and they have an automated slide feeder as an option. In recent years both systems have been adapted also to other formats, namely multiple samples on glass or polyester films.

The MetaSystems CometImager has been around for many years and has been used also to score comets in samples on GelBond® films of the same size as glass slides (personal communication, Dr. G. Koppen, VITO, Belgium). The system presents a gallery of images after scoring, which the operator may scan through quickly to delete atypical comets and artifacts. However, this may introduce a potential for bias.

The authors have participated in the adaptation of the IMSTAR system to 96 minigels on GelBond® films and also 20-well Trevigen glass slides. We score our format of 96 rehydrated/stained minigels (Gutzkow et al., 2013) on films which are either wet (i.e., with a large coverslip covering the total film surface) or semi-dry (i.e., dried for a few hours or days). Some problems in the past in identifying the faint comets (high levels of DNA damage) now seem to be solved. There is little or no difference in the slope of X-ray dose-response curves obtained with the automated (IMSTAR) vs. the manual (Perceptive) system, and the dynamic range is the same. It takes 2–4 h to analyze 96 minigels, implying that a large experiment (four films, 400 samples) can be scored in 1–2 days. Manual scoring would have taken 4–8 days. The Trevigen slides are stained according to the manufacturer’s protocol, i.e., dried samples are rehydrated and stained, using antifade and coverslip (antifade and coverslips may be omitted; A. K. Sharma (Technical University of Denmark, Søborg; personal communication). Eight slides (160 samples) can be scored in a day vs. semi-automated scoring which may take 8 days depending on the number of comets scored per sample.

Automated systems are superior not only in speed but also in avoiding operator-dependent bias. For example, operators tend to select round and undamaged comets in a background of heavily damaged and overlapping comets. In any case, overlapping comets cannot be scored by image analysis, whether automated or not [with a possible exception described in (Wood et al., 2010) for cell aggregates]. This problem may be solved in an automated system by always requiring a certain space next to the comet whether there is a tail there or not. Cell density is critically important in the HT versions. The minigel system should ideally have ∼400 cells per 4–μL minigel. Making parallel samples with different cell numbers is a good option. Trevigen slides should ideally have ∼1000 cells per 30 μL well.

## CONCLUSION AND PERSPECTIVES

Some of the HT systems described here rely on cutting edge technology, whereas others represent minor and low-cost modifications of the original assay. The latter ones are already available commercially or can be introduced into a normal laboratory with little or no need for special equipment. (The authors may be contacted concerning the 12 minigels on glass slides and the 96 minigels on GelBond® film.) It is expected that microwell and fluidic technology will be introduced in future versions of the comet assay. The cost of such systems may be a limitation to their use. Concerning comet scoring, we anticipate that new principles for quantitative determination of the tail magnitude will appear, possibly based on specific staining of single- and double-stranded DNA (Collins et al., 1997b).

The approved protocol for the *in vivo* comet assay which will soon be published by OECD (2013), is based on the traditional comet assay system and does not discuss HT modifications. A consequence of this is that any new version of the comet assay should be validated, at least if intended for use in genotoxicity testing and regulatory toxicology.

## Conflict of Interest Statement

The authors declare that the research was conducted in the absence of any commercial or financial relationships that could be construed as a potential conflict of interest.
